# Microregional RNA sequencing reveals layer-specific smooth muscle cell heterogeneity in human bicuspid aortic valve-associated aortic dilatation

**DOI:** 10.1016/j.jvssci.2026.100444

**Published:** 2026-09-16

**Authors:** Shigeki Koizumi, Chiho Tokunaga, Keiichi Asano, Kenichi Kimura, Hiroko Matsunaga, Eri Motoyama, Hnin Htet Htet Aung, Kaori Sugiyama, Haruko Takeyama, Yuji Hiramatsu, Hiromi Yanagisawa

**Affiliations:** a Life Science Center for Survival Dynamics, TARA, University of Tsukuba, Tsukuba, Japan; b Division of Glocal Pharmacology, Department of Pharmacology, Graduate School of Medicine, The University of Osaka, Osaka, Japan; c Doctoral Program in Medical Sciences, Graduate School of Comprehensive Human Sciences, University of Tsukuba, Tsukuba, Japan; d Department of Cardiovascular Surgery, Saitama Medical Center, Saitama Medical University, Saitama, Japan; e Research Organization for Nano and Life Innovation, Waseda University, Tokyo, Japan; f Center for Spatial Omics Analysis Research, Waseda University, Tokyo, Japan; g Division of Bioinformation and Therapeutic Systems, National Defense Medical College Research Institute, Saitama, Japan; h Graduate School of Advanced Science and Engineering, Waseda University, Tokyo, Japan; i Department of Cardiovascular Surgery, University of Tsukuba, Tsukuba, Japan; j Faculty of Medicine, University of Tsukuba, Tsukuba, Japan

**Keywords:** Aortic diseases, Bicuspid aortic valve disease, Myocyte, smooth muscle, Gene expression profiling

## Abstract

**Objective:**

Smooth muscle cells in the human ascending aorta exhibit layer-specific heterogeneity; however, their molecular profiles and contribution to aortic diseases remain poorly understood. In this study, we applied microregional RNA sequencing to characterize layer-specific transcriptomic signatures and investigate the differences between bicuspid aortic valve (BAV) and tricuspid aortic valve (TAV) aortopathy.

**Methods:**

Dilated ascending aortic wall samples were obtained from nine patients who underwent ascending aorta replacement concomitant with aortic valve replacement: five with BAV and four with TAV. Microdissected tissues were collected from four distinct layers in media using a micro-punching device. RNA was extracted and sequenced, and the transcriptomic differences across the layers and between BAV- and TAV-dilated aortas were analyzed.

**Results:**

Microregional RNA sequencing revealed significant upregulation of several extracellular matrix remodeling genes in the innermost layer, including *FN1*, *VCAN*, *MGP*, and *POSTN*. Among them, *POSTN* was further validated at the protein level, confirming its upregulation in the innermost layer, supporting the validity of this analysis. Next, the transcriptional differences between BAV- and TAV-dilated aortas across individual layers were analyzed. The gene expression differences were the most prominent in the innermost layer. Gene set enrichment analysis revealed that epithelial-mesenchymal transition and transforming growth factor β signaling-related genes were enriched in BAV-dilated aortas. In contrast, inflammation-related genes were enriched in TAV-dilated aortas. We also evaluated the pathways associated with the genes implicated in BAV formation to identify potential upstream mechanisms, revealing that mechanosensing-related genes are coordinately suppressed in BAV-dilated aortas.

**Conclusions:**

The present study demonstrated the application of microregional RNA sequencing to human aortic tissue, revealing layer-specific transcriptomic differences in aortic diseases. The differences in gene-set-level findings indicate distinct biological contexts and mechanisms in BAV- and TAV-dilated aortas, particularly in the innermost media layer. This approach is useful for assessing layer-specific profiles in aortic diseases.

**Clinical relevance:**

This study identified layer-specific heterogeneity within the human aorta, with molecular alterations in the innermost medial layer potentially contributing to differences between TAV- and BAV-associated aortopathy. The findings suggest that layer-specific transcriptome profiles differ across disease types. Accordingly, diagnostic approaches and therapeutic strategies may be more effective when tailored to specific aortic regions and their underlying molecular characteristics.

**Article Highlights**

• **Type of Research:** Human study
• **Key Findings:** Microregional RNA sequencing was successfully applied to human aortic tissue and demonstrated transcriptional differences between the inner and outer layers in the dilated ascending aorta. The layer-specific analysis also identified that the innermost layer showed the most pronounced gene-set-level differences between BAV- and TAV- dilated aorta.
• **Take Home Message:** Microregional RNA sequencing enables comprehensive molecular alteration detection in defined regions in human aortic tissue. This method facilitates the characterization of region-specific profiles, revealing layer heterogeneity and aortic disease-specific alterations.

Tissues and organs are shaped by their microenvironment. The aorta, the largest vessel in the circulatory system, is continuously exposed to pulsatile mechanical forces generated by the cardiac cycle, creating a highly dynamic microenvironment.^1^ To withstand these forces, the human aortic media comprises approximately 50 lamellar units, each containing smooth muscle cells (SMCs) interconnected by extracellular matrix (ECM) components, including elastic fibers. These lamellar structures distribute mechanical stress across the vessel wall through coordinated stretching and recoiling.^2^^,^^3^

In addition to mechanical stimuli, endothelial cells lining the luminal surface are exposed to circulating humoral factors, and these cells communicate with the underlying SMCs to maintain vascular homeostasis. The adventitia, the outermost layer of the vessel wall, provides a niche for fibroblasts, immune cells, vascular progenitor cells, and vasa vasorum; this layer is embedded within collagen-rich ECM, which confers tensile strength and modulates SMC phenotypic state. Under physiological conditions, medial SMCs predominantly exhibit a contractile phenotype; however, they can undergo phenotypic switching toward a synthetic state in response to pathological stimuli, including hypertension, vascular injury, and aortic aneurysm formation.^1^^,^^4^

The SMC phenotype and function are determined by both local microenvironmental cues and their distinct embryonic origins.^5^ Along the longitudinal axis of the aorta, multiple developmental lineages contribute to SMC heterogeneity, including anterior heart field-derived, neural crest-derived, and lateral mesoderm-derived SMCs.^6^, ^7^, ^8^ Notably, lineage-dependent differences in signaling responses, such as transforming growth factor β (TGFβ) signaling, have been reported.^9^ However, definitive molecular markers that distinguish these SMC populations remain incompletely characterized, particularly within the layered structure of the aortic media.^10^ Given this heterogeneity, altered hemodynamic stress arising from disturbed flow and pressure overload may differentially affect spatially distinct SMC populations within the aortic wall.

Bicuspid aortic valve (BAV), a common congenital valve abnormality affecting approximately 1% to 2% of the general population, is a pathological condition that profoundly alters the mechanical microenvironment of the aorta.^11^ Patients with BAV are predisposed to aortic dilatation and aneurysm formation. These vascular complications are often attributed to the abnormal hemodynamic forces generated by eccentric systolic flow.^12^ However, the molecular consequences of these altered mechanical forces on aortic SMCs remain poorly understood. In particular, it is unclear whether SMCs in BAV-associated aortopathy differ from those in tricuspid aortic valve (TAV)-associated disease in a layer-specific manner.

Recently developed transcriptomic approaches, such as bulk RNA sequencing and single-cell (sc) RNA sequencing, have provided important insights into the molecular diversity of vascular SMCs.^13^^,^^14^ However, these methods often lose spatial resolution within the vessel wall. In contrast, spatial transcriptomic technologies are expensive and technically demanding, limiting their application to human tissues. To address this limitation, in this study, we applied a microregional RNA sequencing approach to human aorta samples to define layer-specific transcriptomic signatures for comparing spatial differences between BAV- and TAV-associated aortopathy.

## Methods

### Patients

Nine consecutive patients underwent ascending aorta replacement concomitant with aortic valve replacement for non-genotyped ascending aortic dilatation at the Department of Cardiovascular Surgery, University of Tsukuba Hospital, from 2020 to 2022. Five ascending aortic wall samples from patients with BAV were obtained and analyzed through histopathological and bioinformatic approaches. As controls, four ascending aortic wall samples were obtained from patients who underwent combined ascending aorta replacement and aortic valve replacement for ascending aortic dilatation with TAV dysfunction. The control samples were also included in our previous study.^15^ The study protocol was approved by the Ethics Committee of the University of Tsukuba Hospital (Approval No. H27-217). Written informed consent was obtained from each patient.

### Definition of aortic dilatation and valve disease

Ascending aortic dilatation was defined as an aortic diameter >40 mm and <54 mm,^16^ as measured using preoperative computed tomography. Concomitant ascending aorta replacement during surgery for BAV dysfunction was considered when the ascending aorta diameter exceeded 45 mm following the guidelines of the Japanese Circulation Society.^17^ The surgical indication for all patients was valve dysfunction, such as aortic regurgitation or aortic stenosis. Valve morphology and BAV dysfunction were diagnosed preoperatively using echocardiography, with the final diagnosis confirmed intraoperatively by the surgeons.

### Histology and immunohistochemistry

The collected aortic tissues were fixed with 4% paraformaldehyde, embedded in paraffin, and sectioned at 7-μm thickness. The sections underwent hematoxylin and eosin (H&E) staining for routine histology, Hart’s staining for elastic fibers, Masson trichrome staining for collagen fibers, and Alcian blue staining for glycosaminoglycans using standard histology protocols. For immunohistochemical (IHC) staining, the sections were deparaffinized, rehydrated, and then subjected to antigen retrieval in Tris-ethylenediaminetetraacetic acid (EDTA) buffer (pH 9.0) at 95 °C for 15 minutes, followed by incubation with 3% hydrogen peroxide for 10 minutes and blocking with 5% bovine serum albumin for 1 hour at room temperature. The samples were then incubated overnight at 4 °C with rabbit anti-periostin (1:200, Cat No. ab14041, Abcam) and for 1 hour at room temperature with the biotinylated secondary antibody (1:400, Cat No. ab6720, Abcam). Rabbit IgG isotype control (1:500, Cat No. ab172730, Abcam) was used as a negative control. After amplification with the avidin-biotinylated enzyme complex (Cat No. PK6100, Vector Laboratories), the samples were incubated with 3,3′-diaminobenzidine (DAB) substrate (Cat No. MK210, Takara Bio Inc) for 3 minutes. The samples were counterstained with hematoxylin and mounted with Entellan new. Images were digitally captured with an Axio Imager Z2 (Carl Zeiss), and size adjustment, rotation, and contrast adjustment were performed with Zen Blue (Carl Zeiss) and ImageJ. The mean DAB intensity and DAB-positive area were quntified using ImageJ. The inner and outer edge lines of media were manually drawn, and a centerline equidistant from the two lines was automatically generated by ImageJ. The area between the inner edge and center lines was defined as the “inner side,” and the area between the center and outer edge lines was defined as the “outer side.” Images were processed using the Colour Deconvolution plugin in ImageJ to isolate the DAB signal. The mean DAB intensity and DAB-positive area were measured within the defined regions using a fixed threshold.

### Microregional RNA sequencing

The aortic tissue samples were fixed in 10% neutral buffered formalin overnight at 4 °C and embedded in paraffin. Subsequently, 10-μm cross-sections were prepared and placed onto Kawamoto film (SECTION-LAB). H&E solution was made by mixing pure eosin (diluted 3× in 99.5% EtOH) and M-type New Hematoxylin Solution. We used the microtissue collection device (110-μm diameter) manufactured by Frontier Biosystems, as previously described.^18^ After H&E staining, the punching sites were carefully selected to represent the four distinct layers of the aortic SMCs, including the subluminal side (L1), inner media (L2), medial midline (L3), and the outer media adjacent to the adventitia (L4). L1 was defined as the first 110-μm area from the aortic lumen, excluding subendothelial ECM; L2 was defined as the next 110-μm area adjacent to L1. L3 was defined as the midline of the media, and L4 was defined as the 110-μm area adjacent to the adventitia. Two to three punches were collected from the four layers within the ascending aorta from patients with BAV, and two punches were obtained from those with TAV. Total RNA was extracted using a RNeasy Mini Kit (QIAGEN) or a RNeasy FFPE Kit (QIAGEN) according to the manufacturer’s instructions. RNA was quantified using a Nanodrop One instrument (Thermo Fisher), and cDNA libraries were constructed as previously reported.^19^^,^^20^ Sequencing libraries were prepared using the Nextera XT Kit (Illumina) according to the manufacturer’s instructions. Sequencing was performed on the Illumina Nextseq 2000 platform.

### Data processing and analysis

FASTQ files were analyzed using CLC Genomics Workbench (version 22.0, QIAGEN), and sequences were mapped to the human genome (GRCh38.105). Samples with low protein-coding gene expression and low SMC-marker gene expression, defined as a normalized myosin heavy chain 11 (*MYH11*) expression of <6.0 or a raw count of 0 for other SMC markers, were excluded from further analysis.

Technical replicates for each sample were combined by summing the raw read counts. The count matrix was normalized using the Trimmed Mean of M-values method in the edgeR -package (version 4.4.2) in R software (version 4.4.3, R Foundation for Statistical Computing). Differential gene expression between BAV- and TAV-dilated aorta samples in each layer was evaluated using the edgeR package. For comparisons across the layers in BAV- and TAV-dilated aorta samples, the limma-voom pipeline^21^ (version 3.62.2) was applied to account for within-patient correlations (design ∼ group + patient). Genes with a false discovery rate (FDR) of <0.05 and |log2 fold change| > 1, and raw *P* value < .01 and |log2 fold change| > 1 were considered differentially expressed genes (DEGs) between BAV- and TAV-dilated aorta in each layer and between the inner and outer sides of the TAV- and BAV- dilated aorta, respectively.

Gene Ontology-Biological Process (GO-BP)^22^ and Hallmark^23^ gene sets were obtained from the Molecular Signatures Database (MSigDB, version 2025.1). Over-representative analysis and gene set enrichment analysis (GSEA)^24^ were performed using clusterProfiler (version 4.14.6), and enriched GO-BP (raw *P* value < .01) and Hallmark gene sets (FDR < 0.05) were identified. Gene set variation analysis (GSVA)^25^ was conducted using the GSVA package (version 2.0.7) in R to evaluate the pathway enrichment scores for the Hallmark and target GO-BP gene sets in each sample and layer. Gene set enrichment was evaluated by comparing GSVA scores and rotation gene set testing (ROAST)^26^ using the limma package. Heatmaps were generated using hierarchical clustering based on the Euclidean distance and complete linkage. The formal names of the gene sets analyzed in this study are listed in Supplementary Table I (online only).

Principal component analysis (PCA) plots, volcano plots, and heatmaps were generated using the ggplot2 (version 4.0.0) and pheatmap (version 1.0.13) packages in R. The heatmap, bar graph, and scatter plot of the GSVA score were visualized using Prism 10 (GraphPad, version 10.6.1).

### Statistical analysis

Statistical analyses were conducted using Prism 10, and all data are reported as mean ± standard error of the mean. The Shapiro-Wilk test was used to assess normality. The differences between the inner and outer sides were analyzed using a paired *t* test, and the difference in GSVA scores between the groups within layers was analyzed using Welch’s *t* test. A *P* value of <.05 was considered to indicate significance.

## Results

### Patient characteristics, histology, and microregional RNA sequencing

We obtained dilated ascending aorta samples from patients with TAV (TAV-dilated, n = 4) and BAV (BAV-dilated, n = 5) (Supplementary Table II, online only). Histological analyses showed no significant differences between TAV- and BAV-dilated aortas in terms of the overall structure, elastic fibers, collagen fibers, or glycosaminoglycan distribution (Supplementary Fig 1, online only).

To investigate spatial transcriptomic heterogeneity, we isolated four medial layers (L1-L4) using a micro-punching device (Fig 1). RNA from a total of 92 punched tissue samples was obtained and mapped to the reference genome (GRCh38.105); five samples with low exon-mapping rates were excluded from further analyses. SMC marker gene expression was analyzed in the remaining samples, and four samples with low *MYH11* expression and one additional sample were excluded because the raw counts of both *CNN1* and *TAGLN* were 0 (Supplementary Fig 2, online only). Although we could not entirely rule out possible contamination by non-SMC cells, we confirmed that each sample exhibited high expression of canonical SMC markers (Supplementary Fig 2, online only). Together, these quality-control analyses demonstrated that microregional punching provided the reproducible collection of SMC-enriched samples for spatial transcriptomic analysis.

**Fig 1:**
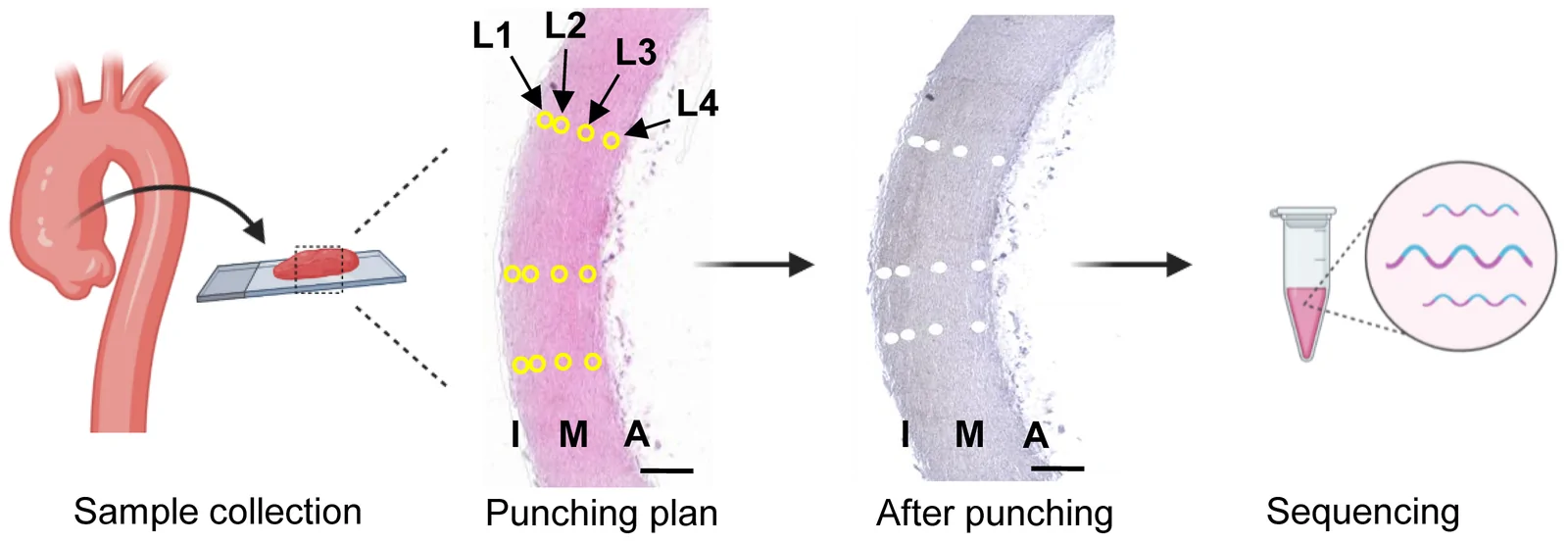
Microregional RNA sequencing in the human aorta. Overview of microregional RNA sequencing and the definition of punching sites. Aortic tissue sections stained with hematoxylin and eosin. Layer L1: 100-μm stripe besides the aortic lumen, excluding the endothelial layer; Layer L2: another 100-μm stripe continuous to Layer L1; Layer L3: 100-μm stripe along the midline; Layer L4: 100-μm stripe adjacent to the adventitia. I: Intima, M: Media, A: Adventitia. Bars are 500 μm. Figures were created using BioRender.

### Layer-specific transcriptional heterogeneity in TAV-dilated aorta

To determine whether microregional RNA sequencing could detect transcriptomic differences across the aortic media, we first analyzed TAV-dilated aortas as a reference to establish layer-resolved transcriptomic profiling. To compare spatial differences, the layers were grouped as inner (inner, L1 and L2) and mid-to-outer (outer, L3 and L4) medial layers. PCA revealed that the samples clustered primarily by patient rather than by medial layer (Fig 2, *A*), indicating that inter-patient variability was the dominant source of transcriptomic variation. Nevertheless, within individual patients, a consistent spatial relationship tended to be maintained according to layer for the samples, suggesting the presence of reproducible layer-associated expression patterns. Therefore, differential expression analyses were performed using a model that accounted for patient-specific effects. Several DEGs were identified between the layers, including *POSTN* (encoding periostin), *MGP* (encoding the matrix gla protein), *VCAN* (encoding versican), *FN1* (encoding fibronectin), and *SERPINE1* (encoding plasminogen activator inhibitor type 1) (Fig 2, *B*). DEGs upregulated on the inner side in the TAV-dilated group were associated with leukocyte chemotaxis and migration, cell-matrix adhesion, and ECM organization (Fig 2, *C*). GSEA further showed that the “epithelial-mesenchymal transition (EMT),” “TNFα signaling via NFκB,” and “inflammatory response” gene sets were upregulated on the inner side (Supplementary Fig 3, *A*, online only).

**Fig 2:**
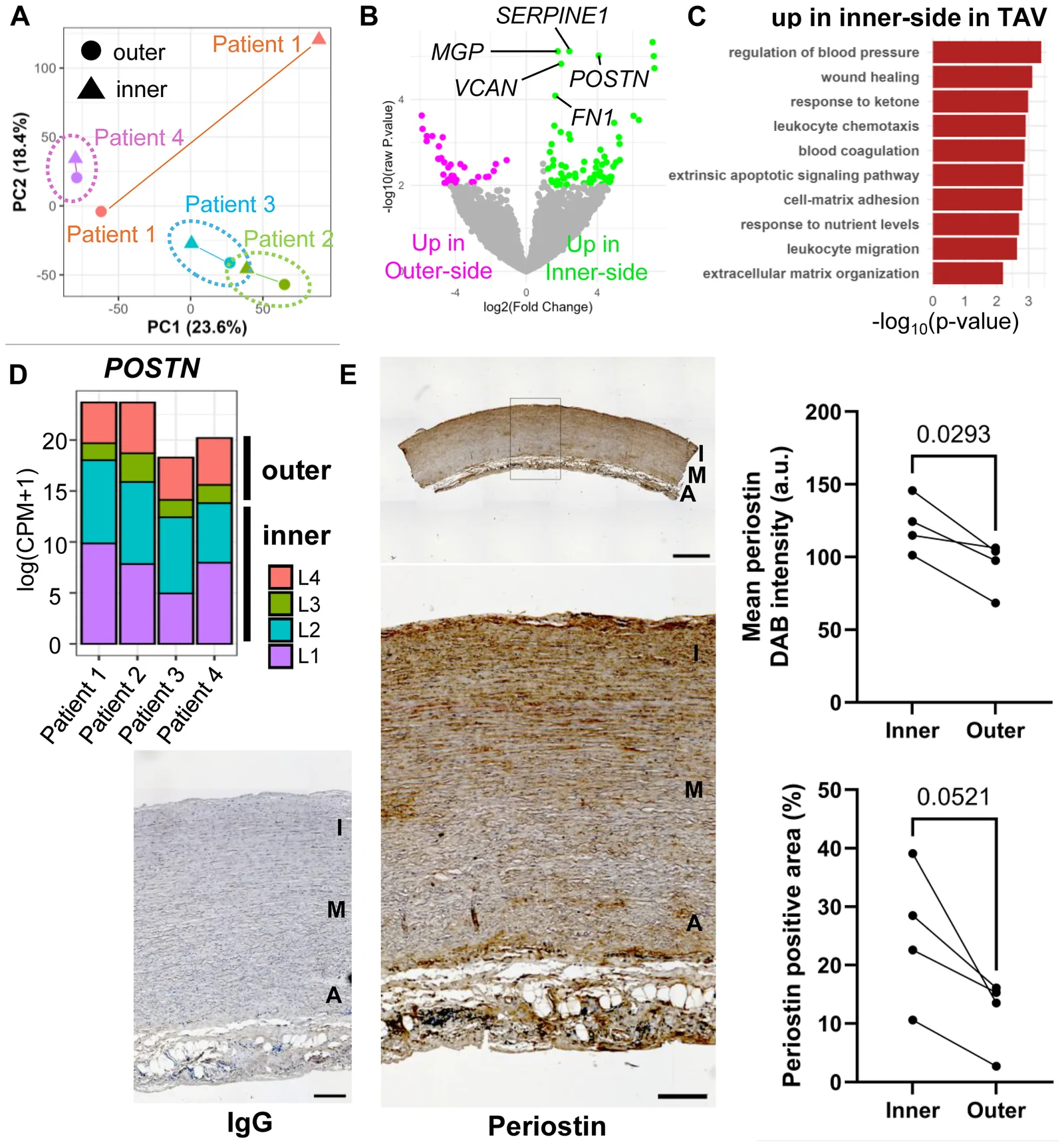
Differential gene expression analysis between the inner side vs outer side and immunohistochemical validation of transcriptomic data. RNA-seq analysis comparing the inner side (Layers L1 and L2) and outer side (Layers L3 and L4) in dilated ascending aortas of patients with tricuspid aortic valve (*TAV*). **A,** Principal component analysis (PCA) plots showing separated groups by patients (*color*) and not by layer (*shape*). **B,** Volcano plots showing upregulated genes (*green*) and downregulated genes (*magenta*) on the inner side (|log2 fold change| > 1, raw *P* value < .01) compared with the outer side. Representative differentially expressed genes are shown. **C,** Gene Ontology-Biological Process (GO-BP) term enrichment on the inner side of TAV-dilated aortas relative to the outer side. **D,** Stacked bar plots showing log (CPM+1) values of *POSTN* in each patient, with stacked segments representing layers. **E,** Representative images of immunohistochemical staining of periostin and IgG control, showing higher protein expression on the inner side of the aorta. Quantification of the mean periostin 3,3′-diaminobenzidine (*DAB*) intensity and periostin-positive area, confirming higher expression on the inner side than on the outer side. Each *dot* represents one patient, and statistical comparisons were conducted through a paired *t* test. I: Intima, M: Media, A: Adventitia. a.u.: arbitrary units. Bars are 1000 μm (*upper*) and 200 μm (*lower*).

Next, we performed IHC to validate the microregional RNA sequencing results and confirm layer heterogeneity at the protein level in the human aorta samples. We focused on periostin, one of the most highly upregulated DEGs between the inner and outer layers of TAV-dilated aortas (Fig 2, *D*). IHC showed higher periostin expression on the inner side, consistent with the transcriptomic data (Fig 2, *E*).

### Distinct transcriptional differences between TAV- and BAV-dilated aortas in the innermost layer (L1)

BAV is associated with aortic dilatation that can progress to aneurysm formation; however, whether altered hemodynamics affect gene expression in SMCs remains unclear. We used a micro-punching approach to detect layer-specific transcriptomic differences between TAV-and BAV-dilated aortas (Fig 3, *A*). Because the results revealed clear layer-specific transcriptional heterogeneity in TAV-dilated aortas, we next examined whether similar regional differences were present in BAV-dilated aortas by comparing the transcriptomes obtained from the inner (L1-L2) and mid-to-outer (L3-L4) layers. PCA showed similar spatial arrangements between the two layers across patients, as observed in TAV-dilated aortas. This finding suggests that regional transcriptomic organization is preserved in BAV-dilated aortas (Supplementary Fig 3, *B*, online only). A patient-adjusted comparison identified several DEGs between the layers, recapitulating the regional differences observed in TAV-dilated aortic media (Supplementary Fig 3, *C*, online only). Eleven genes were commonly upregulated in the L1 and L2 layers of both TAV- and BAV-dilated aortas, most of which are involved in ECM remodeling (Supplementary Fig 3, *D*, online only). DEGs upregulated on the inner side of BAV-dilated aortas were associated with ECM organization, similar to the findings for TAV-dilated aortas, whereas genes related to skeletal and cartilage development and TGFβ signaling were additionally enriched in BAV-dilated aortas (Supplementary Fig 3, *E*, online only). GSEA further revealed that the “EMT” and “TGFβ signaling” gene sets were enriched on the inner side of BAV-dilated aortas (Supplementary Fig 3, *F*, online only).

**Fig 3:**
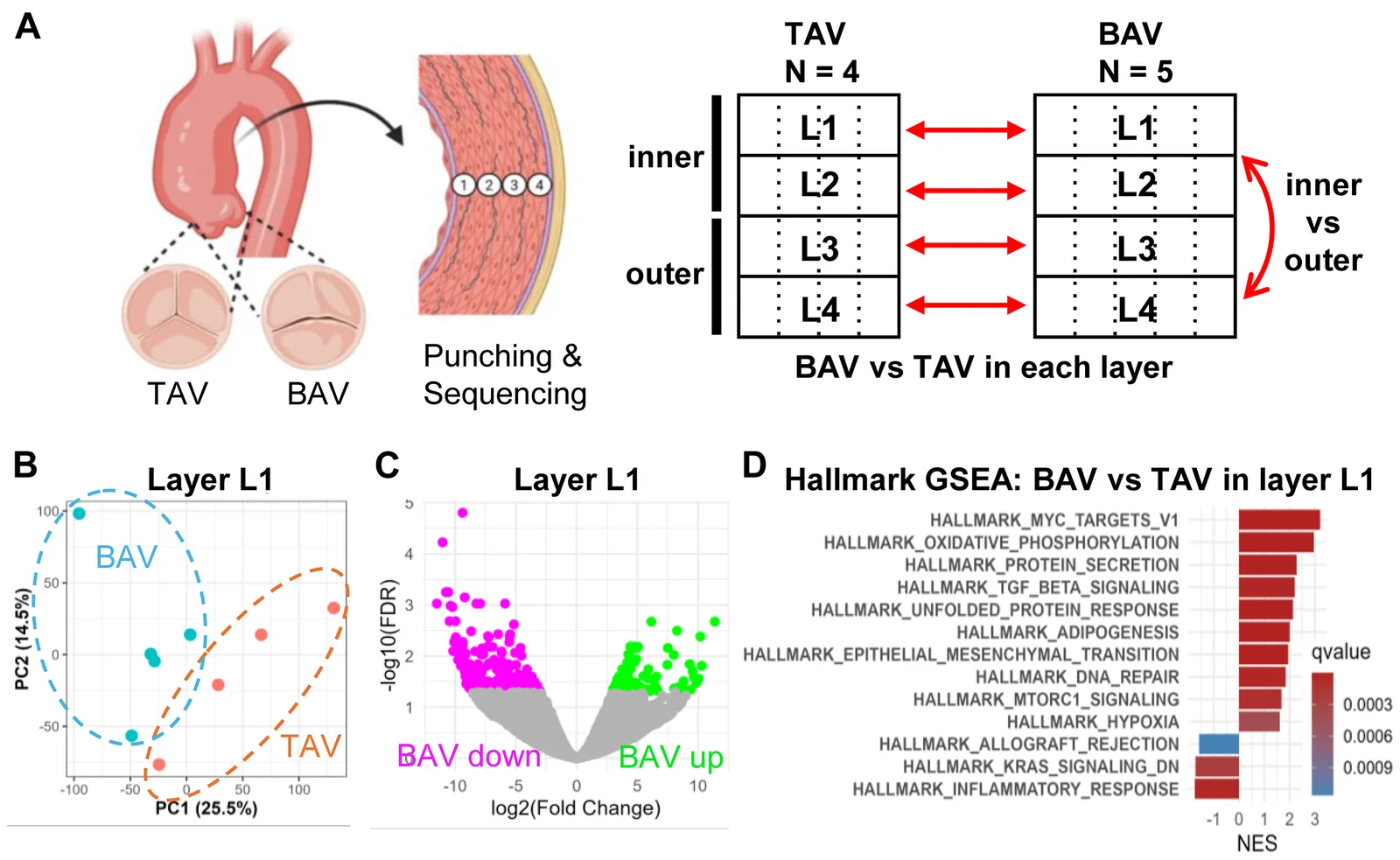
Differential gene expression and gene set analysis between bicuspid aortic valve (*BAV*)- and tricuspid aortic valve (*TAV*)-dilated aortas. RNA-seq analysis comparing TAV and BAV aortic media. **A,** Study design and workflow of analysis. **B,** Principal component analysis (PCA) plots showing separation between the groups in Layer L1 with some variability. **C,** Volcano plots showing upregulated gene (*green*) and downregulated gene (*magenta*) in Layer L1 of BAV aortas (|log2 fold change| > 1, FDR < 0.05) compared with TAV aortas. **D,** Gene set enrichment analysis (*GSEA*) in Layer L1 of BAV aortas relative to TAV aortas. Fig 3, *A* was created using BioRender. *FDR*, False discovery rate; *NES*, Normalized enrichment score.

We next analyzed gene expression in each layer of the aortic media to directly compare BAV- and TAV-dilated aortas. PCA showed separation between the TAV and BAV groups in the innermost layer (Layer L1), although with some variability (Fig 3, *B*). In contrast, no clear separation was observed in the L2 and L4 layers (Supplementary Fig 4, *A*, online only). The highest number of DEGs was found in Layer L1 (Fig 3, *C*, BAV-dilated aortas: 80 genes upregulated [Supplementary Table III, online only]; 248 genes downregulated; data not shown), whereas substantially fewer DEGs were detected in the L2-L4 layers (Supplementary Fig 4, *B*, online only). These findings suggest distinct SMC transcriptional features in Layer L1 in the BAV-dilated aorta from those in TAV.

### Distinct pathway activation in TAV- and BAV-dilated aortas in the innermost medial layer

Next, we performed gene set analysis to determine layer-specific functional differences between TAV- and BAV-dilated aortas. In Layer L1, GSEA using Hallmark gene sets showed significant enrichment of “TGFβ signaling,” “EMT,” and “hypoxia” in BAV, whereas “inflammatory response” was enriched in TAV (Fig 3, *D*; Supplementary Fig 4, *C*, online only). In BAV-dilated aortas, “EMT” remained enriched in Layer L2, and “coagulation” was enriched in Layer L3. No significant enrichment was observed in Layer L4. In contrast, the “inflammatory response” gene set was consistently enriched across Layers L1-L3 in TAV-dilated aortas (Supplementary Fig 4, *D*, online only).

To compare pathway activity across the samples and layers, we performed GSVA and visualized enrichment scores (Fig 4; Supplementary Fig 5, online only). The GSVA scores for EMT and TGFβ signaling pathways were generally higher in BAV-dilated aortas, particularly in Layer L1, with statistical significance for TGFβ signaling and a trend for EMT. In contrast, the GSVA score of inflammatory pathways was higher in TAV-dilated aortas, with a trend observed in Layer L1. The enrichment of EMT in BAV-dilated aortas^27^ and inflammation in TAV-dilated aortas^28^^,^^29^ is consistent with previous reports, supporting our findings. Taken together, these results indicate distinct transcriptional programs between TAV- and BAV-dilated aortas at the pathway level, with the most pronounced differences in the innermost layer (L1).

**Fig 4:**
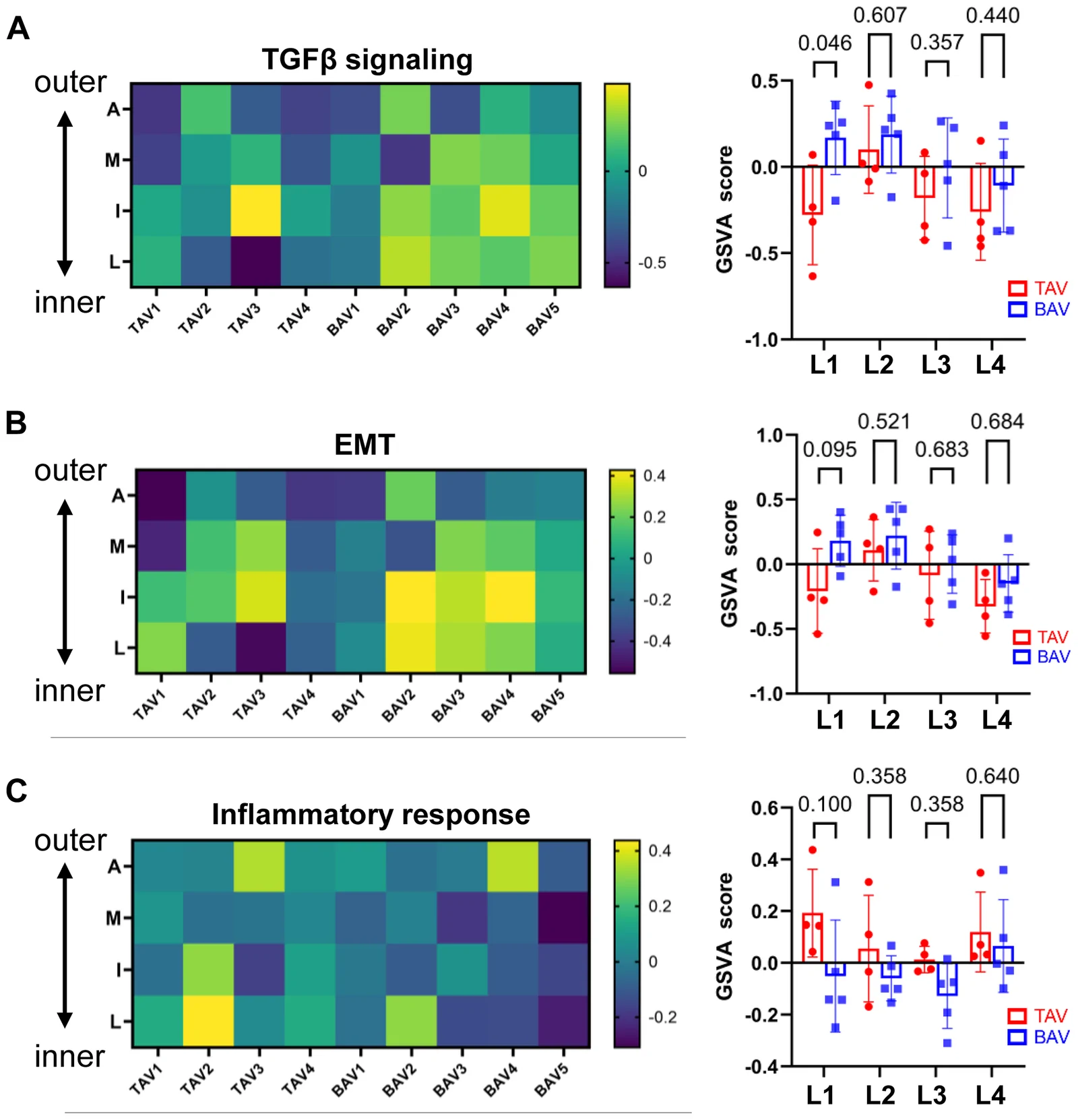
Gene set variation analysis (patient - layer pathway enrichment score). Heatmap and bar plots showing the gene set variation analysis (*GSVA*) scores of the Hallmark gene set: **A**, transforming growth factor β “(*TGFβ*) signaling,” **B**, **“**Epithelial-mesenchymal transition (EMT),” and **C**, “Inflammatory response” across patients and layers. The heatmap shows GSVA pathway enrichment scores for each patient-layer samples, with patients on the x-axis and aortic wall layers on the y-axis. Each *dot* represents one patient, and statistical comparisons were conducted using Welch’s *t* test.

### Mechanosensing-related transcriptional changes in the innermost layer of BAV-dilated aortas

Finally, we investigated candidate upstream pathways that might contribute to the distinct transcriptional features of the innermost layer in BAV-dilated aortas. We first examined whether BAV-associated signaling pathways were enriched in Layer L1 of BAV-dilated aortas. Abnormal mechanical stimuli due to disturbed flow and underlying gene mutations have been implicated in BAV aortopathy.^12^^,^^30^^,^^31^ Thus, we evaluated the GSVA scores for these pathways in Layer L1 (Fig 5, *A*). GSVA suggested the higher activity of the “NOTCH signaling” and “canonical WNT signaling” pathways in BAV-dilated aortas (Supplementary Fig 6, *A*, online only). However, ROAST did not demonstrate the consistent directional upregulation of these pathways (Fig 5, *B*). In contrast, ROAST revealed lower GSVA scores for the “detection of mechanical stimulus” gene set in BAV-dilated aortas (the scores were higher in TAV aortas, Fig 5, *C*), together with significant directional regulation (Fig 5; *B*, Supplementary Fig 6, *B*, online only). This result suggests altered mechanosensing-related transcriptional activity in BAV-dilated aortas.

**Fig 5:**
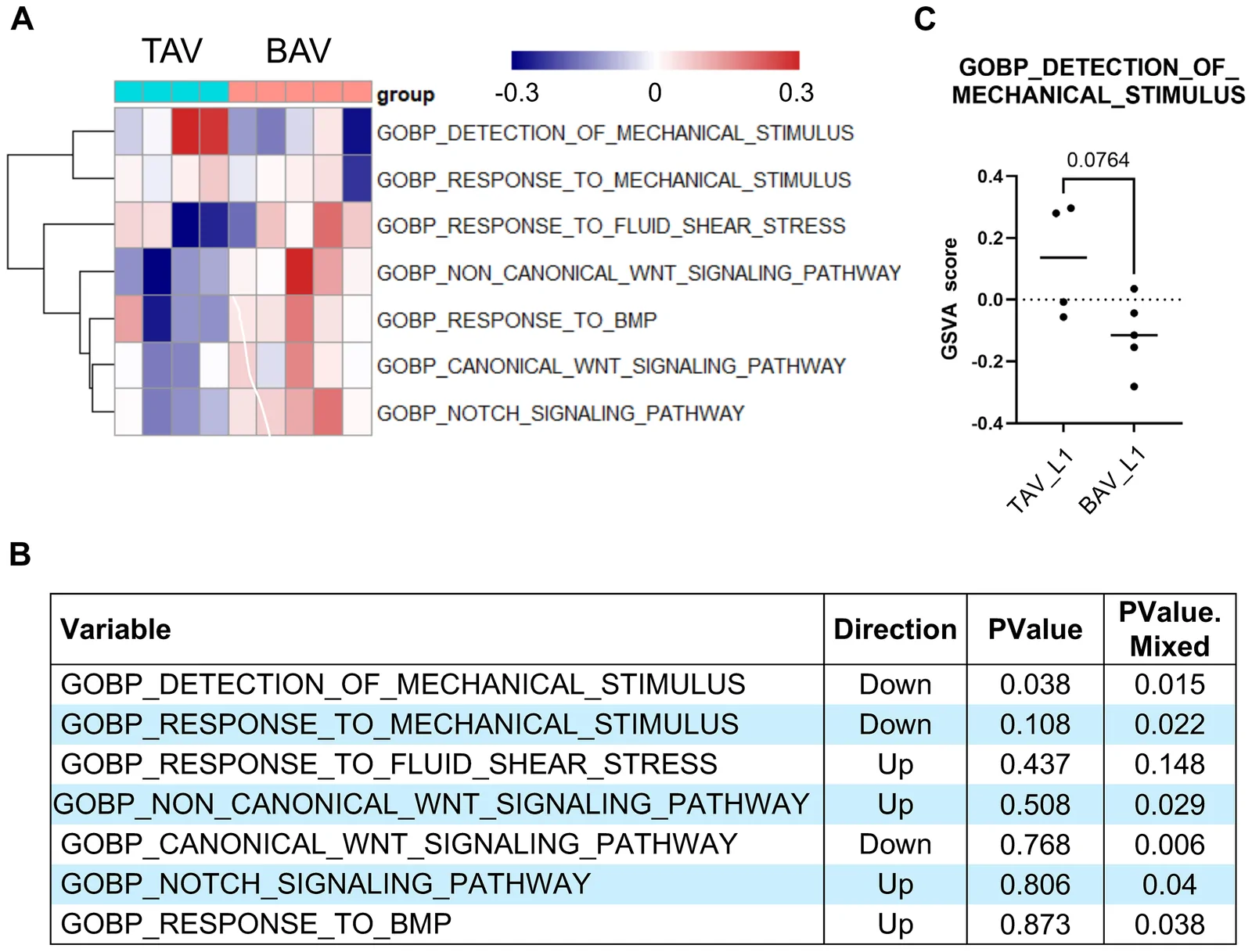
Gene set variation analysis (GSVA) and rotation gene set testing (ROAST) analysis of bicuspid aortic valve (*BAV*)-related gene sets in the innermost layer. **A,** Heatmap showing the unscaled GSVA score of BAV-related gene sets across tricuspid aortic valve (*TAV*) and BAV aortas. **B,** ROAST analysis showing the direction of regulation for BAV-related gene sets, with “Detection of mechanical stimulus” showing consistent downregulation. **C,** Scatter plot showing the GSVA score for “Detection of mechanical stimulus,” with each *dot* representing an individual sample and *horizontal bars* indicating the group means.

## Discussion

In the present study, we applied a simple microregional punching system combined with RNA sequencing to detect molecular differences within predefined regions of the aorta. Although routine histological analysis revealed no significant differences between aortic dilatation associated with TAV or BAV, targeted transcriptomic profiling revealed regional SMC heterogeneity between TAV- and BAV-dilated aortas, especially in the L1 innermost layer.

Several approaches have been developed to investigate regional heterogeneity within the aortic wall. In animal models, SMCs in the proximal aortic media can be distinguished through lineage tracing using Wnt-1-Cre and MEF2C-Cre, which reveal a clear demarcation between the inner and the outer medial layers.^8^ Cre-based labeling combined with cell isolation enables the analyses of region-associated gene expression, but this approach depends on the specificity of Cre drivers and is not applicable to human tissues. In human studies, scRNA sequencing is typically used to identify transcriptionally distinct cell populations and subsequently to infer or validate their spatial localization based on the expression of selected marker genes. Consequently, scRNA sequencing provides detailed insights into cellular heterogeneity, and this sequencing is highly effective for generating cell-driven hypotheses. Recent studies have shown differences in transcriptome profiles and chromatin accessibility between SMCs of distinct developmental origins, indicating that these differences are involved in aortic pathology.^32^^,^^33^ Although these studies have provided valuable insights into cell-origin-specific differences, they have not directly addressed the regional heterogeneity of SMCs.

In contrast, our microregional RNA-sequencing approach preserves the anatomical identity of spatially defined tissue regions without enzymatic dissociation, enabling direct transcriptomic comparison between adjacent aortic microregions while maintaining their native spatial context. Thus, this approach is particularly well-suited for evaluating region-driven hypotheses, such as the regional heterogeneity of vascular SMCs, and it complements single-cell transcriptomic analyses.

Another emerging strategy to preserve spatial information is imaging-based spatial transcriptomics. Mizrak and colleagues applied this technique to the human aneurysmal aorta and visualized the spatial distribution of SMC subtypes.^34^ Their study provided spatially comprehensive information and identified distinct SMC subtypes located in the inner and outer regions, supporting regional SMC heterogeneity. However, using this technique, they examined a limited number of genes in a biased manner, limiting the capacity of the technique to address functional heterogeneity. Our approach combines comprehensive molecular alteration detection with subregional specificity.^18^ Even in human aortic media with abundant ECM, most microdissected tissues could be successfully collected and analyzed while preserving SMC marker expression. Although this method does not achieve single-cell resolution, it provides unbiased transcriptomic profiling within predefined anatomical regions. Thus, this approach complements both scRNA sequencing and image-based spatial transcriptomics by combining comprehensive transcriptome profiling with anatomically defined sampling.

Comparisons between the inner and outer sides of the media revealed a consistent enrichment of ECM remodeling-associated genes involving aortic pathology, such as *FN1*,^35^ *VCAN*,^36^ and *POSTN*,^37^ on the inner side of TAV- and BAV-dilated aortas. *POSTN*, a well-known activated fibroblast marker, shows minimal expression in the normal vasculature; however, vascular injury and mechanical strain induce *POSTN* expression in vascular SMCs, thereby promoting SMC differentiation and migration.^37^ We confirmed *POSTN* enrichment on the inner side of the pathological aorta at the protein level. A previous report showed that periostin was localized to the subendothelial superficial layer of normal cardiac valves.^38^ Because normal control tissues were lacking in the present study, the pathological significance of the inner-side enrichment of periostin in the dilated aorta remains unclear. Nevertheless, the concordance between the transcriptomic and protein-level expression patterns supports the reliability of this technique.

To demonstrate the biological utility of this approach, we next compared transcriptome profiles between BAV- and TAV-dilated aortas. This layer-specific comparison revealed the greatest differences in the innermost layer. This finding appears to be consistent with the currently proposed pathogenesis of BAV aortopathy, in which turbulent flow induced by BAV^12^ and impaired responses to mechanical stress due to gene mutations^27^ lead to endothelial dysfunction and consequently alter the SMC phenotype and differentiation.^39^^,^^40^

In addition, this approach has the advantage of enabling gene-set-level analysis, which is considered to provide more biologically reliable and interpretable findings than single-gene analysis.^41^ The analysis of the innermost layer revealed that TGFβ- and EMT-related gene sets were enriched in BAV-dilated aortas, whereas inflammation-related gene sets were enriched in TAV-dilated aortas. Abnormal TGFβ signaling is a well-known cause of TAA and BAV aortopathy.^42^^,^^43^ A previous report demonstrated decreased TGFβ bioavailability and compromised TGFβ signaling in BAV aortopathy.^44^ In contrast, our analysis showed higher GSVA scores for TGFβ-related gene sets, highlighting the complexity of TGFβ signaling in BAV aortopathy.

The enrichment of an immune response in TAV-dilated aorta is consistent with the results of the ASAP study, which generated the largest microarray data for comparing BAV- and TAV-associated aorta.^29^ Recently, two research centers reported that aortic regurgitation causes abnormal intimal atherosclerosis and endothelial inflammation, which lead to macrophage infiltration into the media.^45^^,^^46^ Because our data showed that more patients with TAV had aortic regurgitation than those with BAV, the enrichment of an inflammatory response in the innermost layer of the TAV-dilated aorta may reflect the inflammatory nature of aortic regurgitation. Although these differences have also been reported in bulk tissue studies, our approach enables gene-set-level analyses that reveal candidate upstream regulatory pathways and identify subtle regional specific differences that could be obscured in conventional bulk RNA sequencing.

Finally, we used GSVA scores analyzed with *t* tests and ROAST testing to assess gene set enrichment in the two pathologies; GSVA score analysis focuses on the biological relevance of gene set activity, and the ROAST testing evaluates whether genes within a given gene set show consistent directional changes. These analyses showed a consistent downward trend for the genes related to the detection of mechanical stress, including some ion channels, in BAV-dilated aortas, and the enrichment score also tended to be lower in BAV-dilated aortas. Given that various gene mutations associated with ion channels, such as *KCNJ2* and *KCNH2*,^47^^,^^48^ have been implicated in aortic valve malformation, including BAV, abnormal mechanical sensing may underlie the pathophysiology of BAV aortopathy, and further validation of this hypothesis is warranted to establish mechanistic causality.

Overall, the approach reported in this study enables gene-set-level analysis while preserving the spatial context, thereby facilitating the generation of hypotheses regarding region-specific biological differences within localized areas. As another application, given its cost-effectiveness, the method can be effectively combined with imaging-based spatial transcriptomics techniques, which provide high spatial coverage but limited depth. Furthermore, the dissected tissues are suitable for proteomic analysis, potentially enabling the investigation of the relationship between gene expression and ECM composition at the microregional level.^18^ This approach may also facilitate the identification of anatomically vulnerable layers in various aortic diseases; thus, it can contribute to translational research by providing insights into layer-specific therapeutic targets.

### Limitations

This study has several limitations. First, the sample size was limited to four patients with TAV and five patients with BAV, and the sample size may have been insufficient for robust analyses of human tissue with biological variability. With this sample size, subtle differences at the transcriptome or gene-set-level should be interpreted carefully to avoid overinterpretation. Second, the patients' backgrounds varied. Notably, imbalanced sex proportions and the prevalence of aortic valve deterioration (aortic stenosis or aortic regurgitation) may affect aortic tissue vulnerability and mechanical stress, potentially confounding the observed findings. In addition, although various gene mutations are implicated in BAV aortopathy, the genetic background was not assessed. Third, the lack of normal aortic tissue as a control limits the interpretation of our results. Future studies involving large cohorts with appropriate controls are necessary to evaluate the robustness of these findings.

## Conclusions

The present study demonstrates that our microregional RNA sequencing approach enables the layer-specific, gene-set-level analysis of the human aorta, revealing differences across layers and aortic diseases. Transcriptional differences between BAV- and TAV-dilated aortas were the most pronounced in the innermost layer, suggesting that this region plays a key role in distinguishing the two conditions. This approach is valuable for assessing layer-specific molecular profiles in aortic diseases.

## Availability of data and materials

The data presented in this study are available upon request from the corresponding authors. The sequencing data for patients with BAV are stored in Gene Expression Omnibus (GEO) repository, https://www.ncbi.nlm.nih.gov/geo/, accession number GSE329454. The control sequencing data (patients with TAV) used in this study were obtained from our previously published dataset (GSE 318877).^15^

## Author Contributions

Conception and design: HY, KK, CT

Analysis and interpretation: SK, CT, KA, KK, HM, KS, HY

Data collection: SK, CT, KK, HM, EM, HA, KS, HT, YH

Writing the article: SK, CT, KA, KK, YH, HY

Critical revision of the article: CT, KA, KK, HM, EM, HA, KS, HT, YH, HY

Final approval of the article: SK, CT, KA, KK, HM, EM, HA, KS, HT, YH, HY

Statistical analysis: SK

Obtained funding: CT, HT, HY

Overall responsibility: HY

YH and HY contributed equally to this article as co-senior authors.

## Declaration of generative AI and AI-assisted technologies in the writing process

During the preparation of this work, the authors used ChatGPT in order to assist with language clarity and readability. After using this tool, the authors reviewed and edited the content as needed and take full responsibility for the content of the publication. In addition, the visual abstract and figures were created using BioRender (agreement number: CI29NJINFH).

## Disclosures

None.

## Figures and Tables

**Supplementary Fig 1 (online only):**
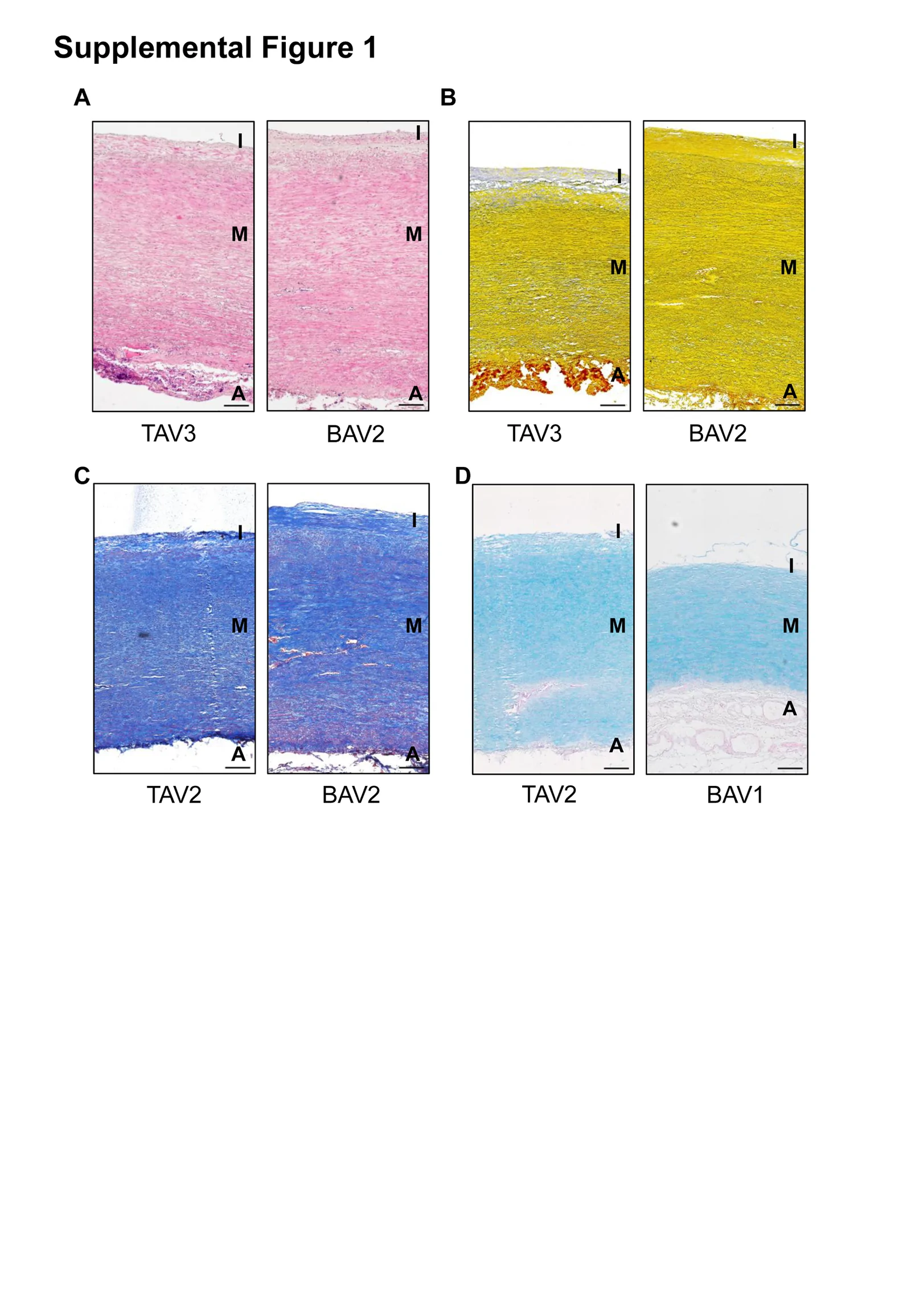


**Supplementary Fig 2 (online only):**
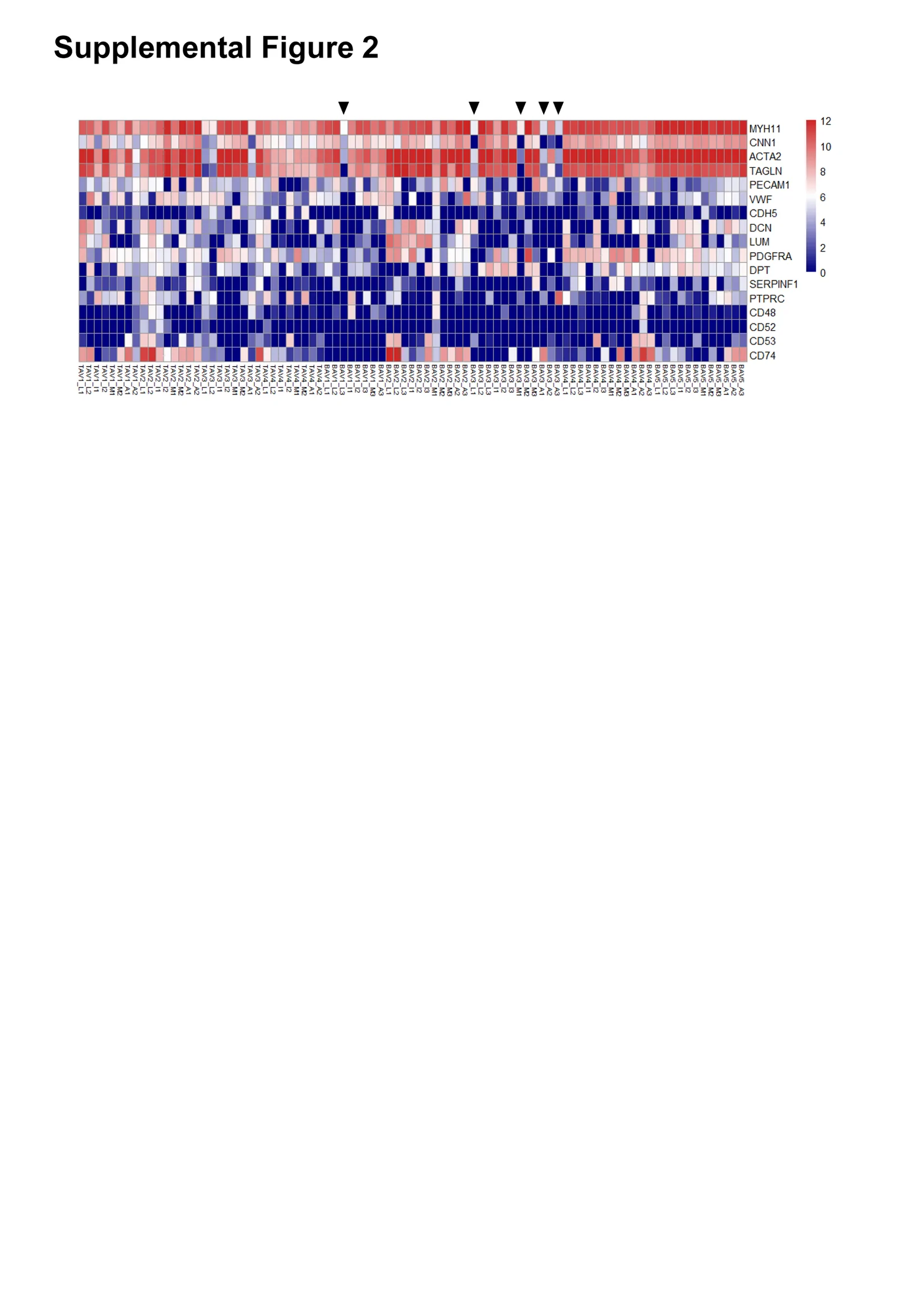


**Supplementary Fig 3 (online only):**
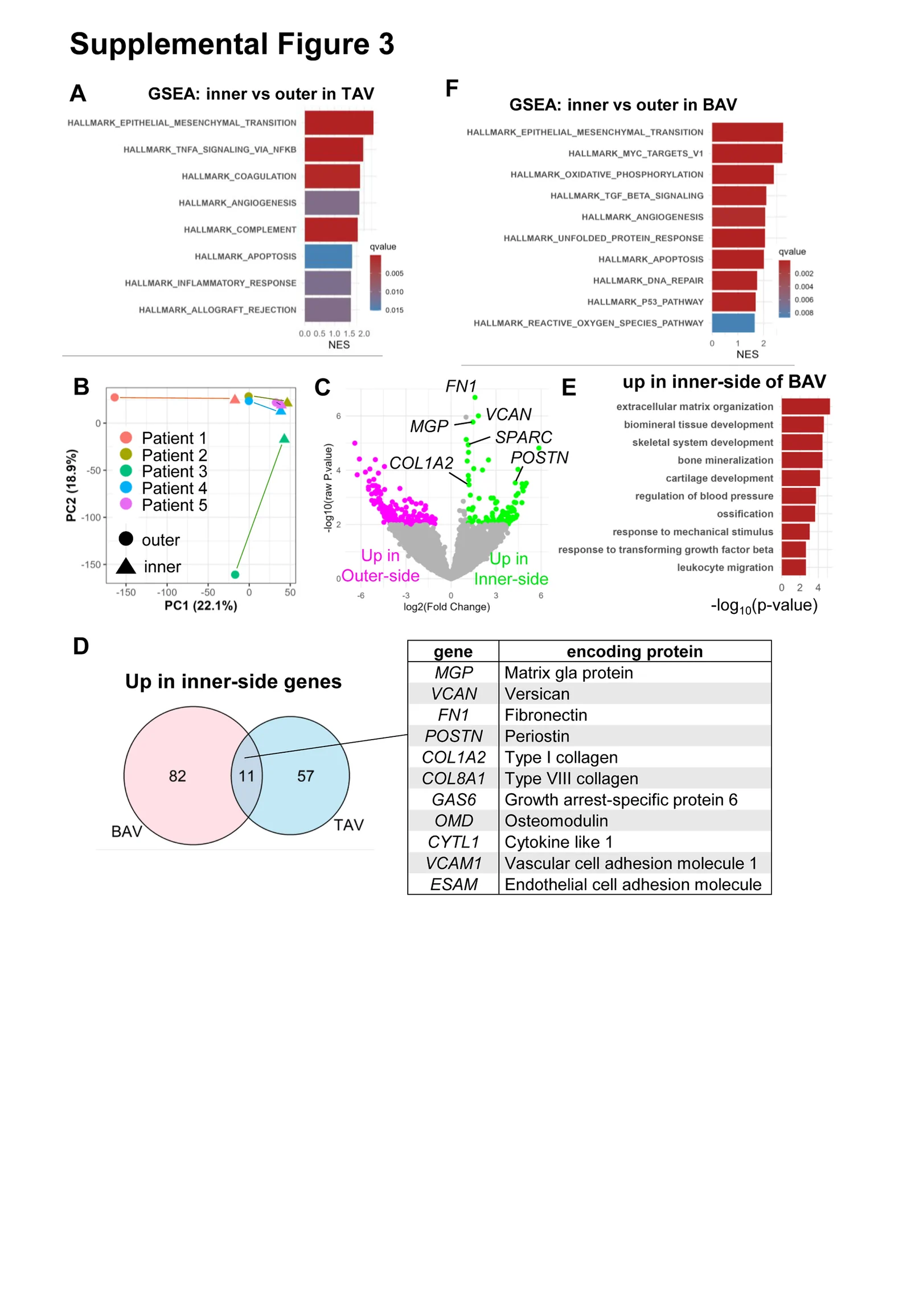


**Supplementary Fig 4 (online only):**
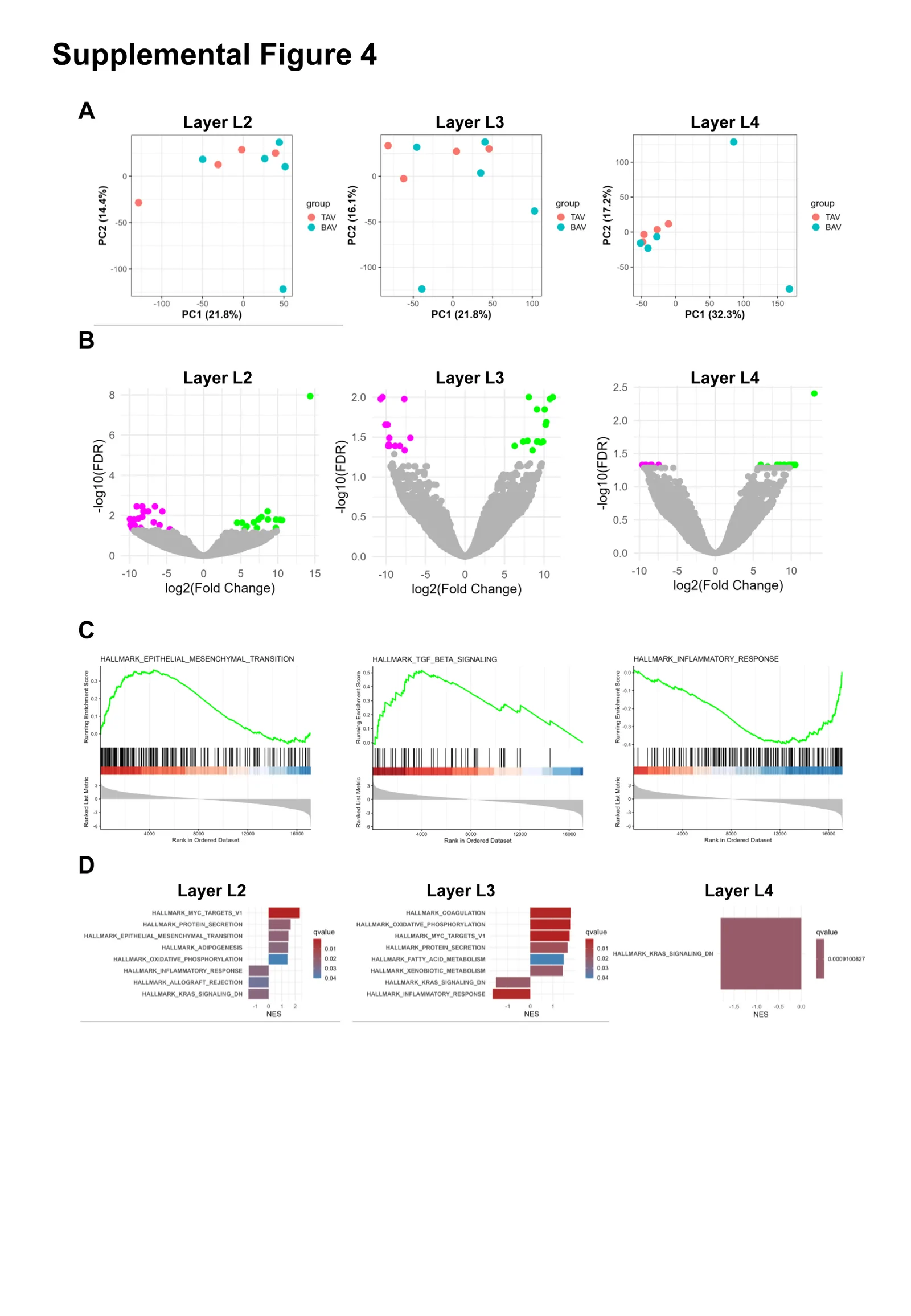


**Supplementary Fig 5 (online only):**
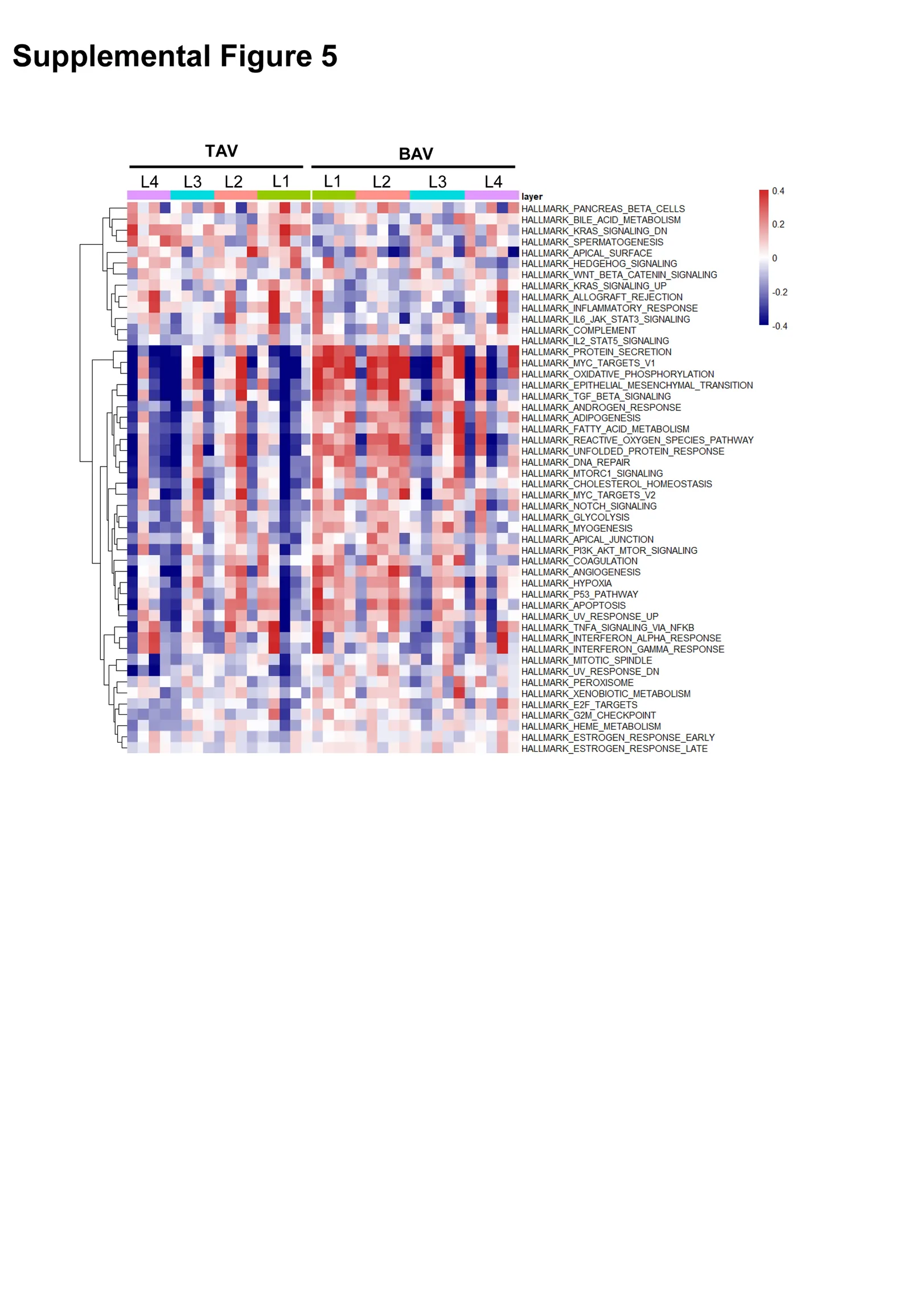


**Supplementary Fig 6 (online only):**
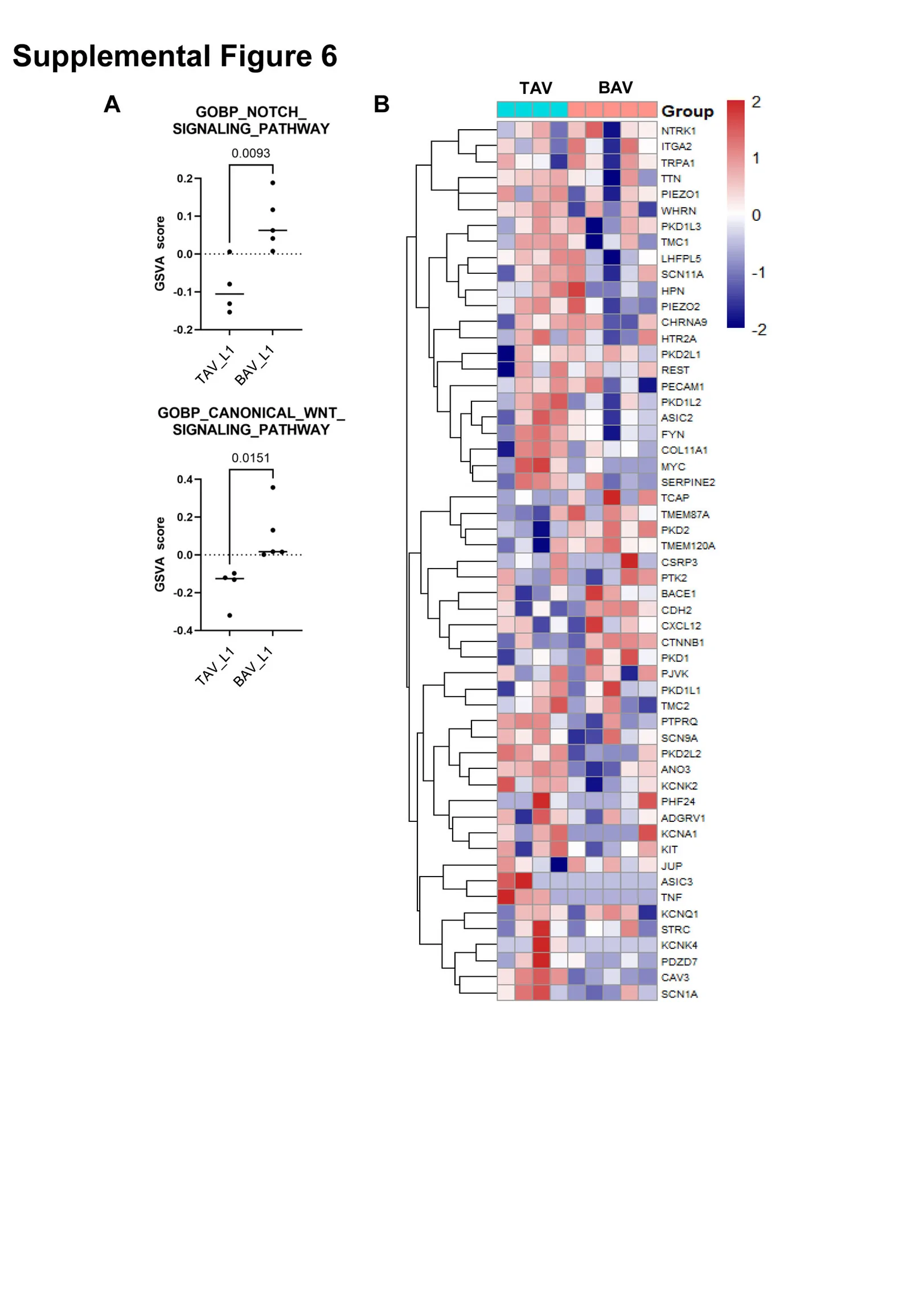


## Appendix (online only)

Supplementary Tables I-III (online only)
