# Supplementary material for: Microregional RNA sequencing reveals layer-specific smooth muscle cell heterogeneity in human bicuspid aortic valve-associated aortic dilatation
Source: JVS Vasc Sci. 2026 Sep 16;7:100444. doi: 10.1016/j.jvssci.2026.100444 (PMC13683236; doi:10.1016/j.jvssci.2026.100444)
Supplement: Supplementary file 1 [file mmc1.docx]

Supplemental Table I. List of gene sets used in this study.

| Abbreviation | Formal gene set name | Database/ID |
| --- | --- | --- |
| "Epithelial mesenchymal transition (EMT)" | HALLMARK_EPITHELIAL_MESENCHYMAL_  TRANSITION | MSigDB Hallmark |
| “TNFα signaling via NFKB” | HALLMARK_TNFA_SIGNALING_VIA_NFKB | MSigDB Hallmark |
| “Inflammatory response” | HALLMARK_INFLAMMATORY_RESPONSE | MSigDB Hallmark |
| “TGFβ signaling” | HALLMARK_TGF_BETA_SIGNALING | MSigDB Hallmark |
| “Hypoxia” | HALLMARK_HYPOXIA | MSigDB Hallmark |
| “Myc_targets_V1” | HALLMARK_MYC_TARGETS_V1 | MSigDB Hallmark |
| “Coagulation” | HALLMARK_COAGULATION | MSigDB Hallmark |
| "NOTCH signaling pathway" | GOBP_NOTCH_SIGNALING_PATHWAY | GO:0007219 |
| "Canonical WNT signaling pathway" | GOBP_CANONICAL_WNT_SIGNALING_  PATHWAY | GO:0060070 |
| “Detection of mechanical stimulus” | GOBP_DETECTION_OF_MECHANICAL_  STIMULUS | GO:0050982 |

Supplemental Table II. Patient information.

| Group | Variable | Gender | Age | Valve dysfunction | Valve morphology | AA  (mm) | Valsalva  (mm) | AR grade | AS grade | Surgery |
| --- | --- | --- | --- | --- | --- | --- | --- | --- | --- | --- |
| TAV | TAV1 | F | 46 | AR | TAV | 47 | 56 | Severe | None | Root replacement |
|  | TAV2 | M | 58 | AR | TAV | 42 | 47 | Severe | None | Root replacement |
|  | TAV3 | F | 74 | AR | TAV | 43 | 39 | Moderate | None | AVR+AAR |
|  | TAV4 | F | 72 | AR | TAV | 50 | 40 | Severe | None | AVR+AAR |
| BAV | BAV1 | M | 52 | AR | Type I  LR/N | 41 | 45 | Severe | None | AVR+AAR |
|  | BAV2 | M | 71 | AS | Type 0 | 54 | 35 | None | Severe | AVR+AAR |
|  | BAV3 | F | 80 | AS | Type II LN/RN | 47 | 30 | Mild | Severe | AVR+AAR |
|  | BAV4 | M | 66 | AS | Type 0 | 46 | 37 | Trace | Severe | AVR+AAR |
|  | BAV5 | M | 48 | ASR | Type II LR/RN | 49 | 39 | Severe | Severe | AVR+AAR |

TAV: Tricuspid aortic valve, BAV: Bicuspid aortic valve, AR: Aortic regurgitation, AS: Aortic stenosis, L: Left coronary cusp, R: Right coronary cusp, N: non-coronary cusp, AA: Ascending aorta, AVR: Aortic valve replacement, AAR: Ascending aorta replacement

Supplemental Table III. DEGs upregulated in BAV patients in layer L1.

| gene | logFC | logCPM | LR | raw p.value | FDR |
| --- | --- | --- | --- | --- | --- |
| FIBIN | 6.152 | 8.545 | 22.834 | 1.77E-06 | 0.002111 |
| CARTPT | 11.383 | 5.855 | 22.820 | 1.78E-06 | 0.002111 |
| SOST | 8.265 | 4.929 | 21.793 | 3.04E-06 | 0.003180 |
| gene:ENSG00000258790 | 10.189 | 6.377 | 20.709 | 5.35E-06 | 0.004136 |
| CTPS1 | 4.795 | 6.549 | 19.145 | 1.21E-05 | 0.006476 |
| ADH5 | 4.424 | 7.286 | 19.105 | 1.24E-05 | 0.006476 |
| CFL2 | 5.060 | 6.461 | 19.019 | 1.29E-05 | 0.006583 |
| KBTBD7 | 7.514 | 5.612 | 18.684 | 1.54E-05 | 0.006911 |
| SNRPE | 4.970 | 7.288 | 18.624 | 1.59E-05 | 0.006911 |
| ATP5MK | 4.582 | 7.865 | 18.412 | 1.78E-05 | 0.007364 |
| CHMP5 | 4.255 | 7.411 | 17.595 | 2.73E-05 | 0.009199 |
| GBP1 | 8.004 | 5.676 | 17.502 | 2.87E-05 | 0.009459 |
| NGRN | 4.908 | 6.944 | 17.033 | 3.67E-05 | 0.011470 |
| RNASE4 | 4.734 | 6.620 | 16.646 | 4.51E-05 | 0.013145 |
| SERBP1 | 4.152 | 8.267 | 16.574 | 4.68E-05 | 0.013432 |
| ADRA2C | 9.311 | 3.834 | 16.223 | 5.63E-05 | 0.014319 |
| ZBTB33 | 7.471 | 5.370 | 16.083 | 6.06E-05 | 0.014809 |
| MSTN | 10.304 | 4.793 | 15.816 | 6.98E-05 | 0.015456 |
| SPARCL1 | 3.751 | 10.930 | 15.270 | 9.32E-05 | 0.018041 |
| PNMA1 | 4.447 | 6.678 | 15.265 | 9.34E-05 | 0.018041 |
| HTATSF1 | 5.914 | 5.336 | 15.240 | 9.47E-05 | 0.018041 |
| PCDHB6 | 9.554 | 4.061 | 15.058 | 0.000104 | 0.019535 |
| GORASP1 | 5.492 | 5.211 | 14.400 | 0.000148 | 0.024587 |
| BCL2L2-PABPN1 | 6.578 | 4.272 | 14.318 | 0.000154 | 0.024973 |
| UQCRH | 3.595 | 7.251 | 14.314 | 0.000155 | 0.024973 |
| LAPTM4A | 3.489 | 8.529 | 14.282 | 0.000157 | 0.024973 |
| CSRP2 | 4.204 | 6.558 | 14.251 | 0.000160 | 0.024973 |
| RANBP6 | 9.641 | 4.151 | 14.201 | 0.000164 | 0.025427 |
| ACKR4 | 5.891 | 5.643 | 13.927 | 0.000190 | 0.027400 |
| KCNA3 | 10.033 | 4.529 | 13.915 | 0.000191 | 0.027400 |
| FBLN5 | 3.333 | 9.428 | 13.757 | 0.000208 | 0.029100 |
| PCYOX1 | 3.773 | 6.697 | 13.498 | 0.000239 | 0.030995 |
| TES | 3.878 | 8.729 | 13.448 | 0.000245 | 0.030995 |
| TCEAL8 | 4.476 | 6.310 | 13.442 | 0.000246 | 0.030995 |
| PET117 | 8.808 | 3.351 | 13.389 | 0.000253 | 0.030995 |
| LEAP2 | 9.870 | 4.366 | 13.167 | 0.000285 | 0.032716 |
| AKAP17A | 4.892 | 5.707 | 13.095 | 0.000296 | 0.033161 |
| PGRMC1 | 3.923 | 7.209 | 13.010 | 0.000310 | 0.033735 |
| DPM1 | 4.544 | 5.445 | 13.005 | 0.000311 | 0.033735 |
| ALDH1B1 | 3.825 | 6.653 | 12.874 | 0.000333 | 0.034402 |
| SNRPB2 | 4.347 | 6.473 | 12.765 | 0.000353 | 0.034739 |
| SLU7 | 3.982 | 6.109 | 12.754 | 0.000355 | 0.034739 |
| SGCB | 3.718 | 6.880 | 12.559 | 0.000394 | 0.036071 |
| CD164 | 3.205 | 7.952 | 12.508 | 0.000405 | 0.036071 |
| TMED4 | 3.779 | 7.383 | 12.497 | 0.000408 | 0.036071 |
| MFF | 3.723 | 7.246 | 12.482 | 0.000411 | 0.036071 |
| ATP5PB | 4.397 | 5.917 | 12.378 | 0.000434 | 0.036950 |
| PNRC2 | 3.749 | 6.382 | 12.371 | 0.000436 | 0.036950 |
| SERF1A | 6.419 | 4.346 | 12.360 | 0.000439 | 0.036999 |
| SLC22A3 | 3.501 | 7.461 | 12.211 | 0.000475 | 0.038852 |
| VAMP4 | 3.828 | 6.631 | 12.208 | 0.000476 | 0.038852 |
| SYNM | 3.912 | 6.093 | 12.150 | 0.000491 | 0.039058 |
| RNF5 | 5.248 | 5.029 | 12.090 | 0.000507 | 0.040106 |
| HRCT1 | 6.521 | 4.190 | 12.031 | 0.000523 | 0.040731 |
| C6orf226 | 8.389 | 2.961 | 12.004 | 0.000531 | 0.040731 |
| SRSF8 | 4.866 | 5.506 | 12.002 | 0.000531 | 0.040731 |
| MORF4L2 | 3.208 | 8.604 | 11.992 | 0.000534 | 0.040731 |
| KCTD9 | 3.796 | 6.380 | 11.961 | 0.000543 | 0.040803 |
| TMED5 | 3.520 | 6.914 | 11.877 | 0.000568 | 0.041200 |
| POLR2K | 4.553 | 5.801 | 11.876 | 0.000568 | 0.041200 |
| MYL12B | 3.234 | 8.232 | 11.825 | 0.000584 | 0.041200 |
| gene:ENSG00000280128 | 8.033 | 4.006 | 11.691 | 0.000628 | 0.042839 |
| CDC42EP3 | 3.001 | 7.513 | 11.607 | 0.000657 | 0.043631 |
| ZNF302 | 5.260 | 5.037 | 11.607 | 0.000657 | 0.043631 |
| gene:ENSG00000279821 | 8.637 | 3.191 | 11.560 | 0.000674 | 0.043948 |
| ZNF396 | 5.746 | 4.642 | 11.537 | 0.000682 | 0.044306 |
| TCEAL5 | 8.803 | 3.343 | 11.506 | 0.000694 | 0.044729 |
| SFRP1 | 4.077 | 8.725 | 11.499 | 0.000696 | 0.044746 |
| FABP7 | 9.405 | 3.916 | 11.469 | 0.000708 | 0.044774 |
| COPS9 | 3.918 | 5.883 | 11.402 | 0.000734 | 0.045631 |
| NEXN | 3.200 | 8.785 | 11.349 | 0.000755 | 0.046485 |
| LEPROT | 3.327 | 7.251 | 11.282 | 0.000782 | 0.046990 |
| CNBP | 3.157 | 8.303 | 11.247 | 0.000797 | 0.047311 |
| SRP9 | 3.013 | 7.507 | 11.219 | 0.000810 | 0.047408 |
| ORMDL1 | 3.394 | 6.701 | 11.173 | 0.000830 | 0.047607 |
| MRPL57 | 4.684 | 6.599 | 11.164 | 0.000834 | 0.047607 |
| FAM50B | 8.362 | 2.945 | 11.160 | 0.000836 | 0.047607 |
| MAK | 6.684 | 5.805 | 11.157 | 0.000837 | 0.047607 |
| gene:ENSG00000228144 | 6.031 | 4.910 | 11.092 | 0.000867 | 0.048477 |
| CPE | 3.175 | 9.855 | 11.058 | 0.000883 | 0.048650 |
